# Dispositional Mindfulness and Post-traumatic Stress Symptoms in Emergency Nurses: Multiple Mediating Roles of Coping Styles and Emotional Exhaustion

**DOI:** 10.3389/fpsyg.2022.787100

**Published:** 2022-03-22

**Authors:** Yuan Yuan, Zonghua Wang, Yanxia Shao, Xia Xu, Fang Lu, Fei Xie, Wei Sun

**Affiliations:** ^1^First Affiliated Hospital, Army Medical University, Chongqing, China; ^2^School of Nursing, Army Medical University, Chongqing, China; ^3^Department of Nursing, Army Medical Center of PLA, Chongqing, China

**Keywords:** emergency department, nurses, dispositional mindfulness, PTSD, coping styles, multiple mediation model

## Abstract

**Objective:**

To explore the relationships between dispositional mindfulness (DM) and their post-traumatic stress symptoms (PTSS) of emergency nurses, and the mediating effects of coping styles and emotional exhaustion (EE).

**Methods:**

A cross-sectional survey study was conducted to collect data on DM, coping styles, EE, and PTSS among 571 emergency nurses from 20 hospitals in Chongqing, China. Correlation and structural equation models (SEMs) were used to evaluate the relationship among variables.

**Results:**

Emergency nurses with lower dispositional mindfulness, higher emotional exhaustion and preference for negative coping (NC) revealed more PTSS. The effect of NC on PTSS was partially mediated by emotional exhaustion. Negative coping and emotional exhaustion played concurrent and sequential mediating roles between dispositional mindfulness and PTSS.

**Conclusion:**

This study has made a significant contribution to existing literature. It was suggested to develop interventions aimed at enhancing mindfulness, reducing negative coping strategies, and alleviating emotional exhaustion, which may be effective at reducing or alleviating post-traumatic stress symptoms of emergency nurses.

## Introduction

Emergency nurses are routinely exposed to work-related traumatic events in their practice, such as severe injuries, death, suicide, and suffering, and also frequently encounter verbal and physical aggression from patients and their families (Gillespie et al., 2013; Guan et al., 2019). These direct and indirect traumas may result in significant psychological consequences; importantly, emergency nurses have little time for recovery due to the emergency department’s hectic pace (Gates et al., 2011). Emergency nurses are at high risk of post-traumatic stress symptoms (PTSS) when they experience repetitive exposure to uncontrolled, trauma situations. As previously reported, approximately 57.2 ~ 97.0% of emergency nurses have at least one PTSS; such findings are consistent across various countries, and approximately 8.5 ~ 25.0% of these nurses meet clinical post-traumatic stress disorder (PTSD) criteria (Adriaenssens et al., 2012; Kim and Yeo, 2020), which was higher than the PTSD prevalence rates reported among the general population (6.8%) and nurses from other departments (6.7 ~ 13.0%; Kessler et al., 2005; Zerach and Shalev, 2015; Colville et al., 2017). PTSS may manifest in the form of exaggerated, startled responses, irritability, nightmares, sleeping difficulties, loss of concentration, and lack of interest in daily life (Beck et al., 2017). These psychosomatic distresses may reduce the ability of nurses to efficiently manage patients and increase the probability of nursing errors and sick leave (Sendler et al., 2016; Salmon and Morehead, 2019). Thus, understanding PTSD symptoms’ influencing factors and the associated, psychological-care interventions might be beneficial for emergency nurses, which is of great significance.

Over the past few years, mindfulness has received considerable attention in traumatic-related studies and has shown a close relationship with psychological well-being (Thompson et al., 2011; Chen et al., 2020). Mindfulness is often described as “bring[ing] one’s complete attention to the experiences occurring in the present moment, in a non-judgmental or accepting way” (Baer et al., 2016). Present-oriented conscious attention and non-judgmental attitudes are key elements of mindfulness, which are regarded as effective antidotes against psychological distress (Keng and Tong, 2016). When experiencing difficult and emotional events, mindful individuals take perspective, calmly and objectively evaluate their feelings, and confront their emotions in a more effective manner, rather than avoid or become overwhelmed (Hülsheger et al., 2013). The separation between the ego and internal/external events may help to decrease trauma-related emotions and physical reactions. An et al. (2019) found that Chinese adolescents who were high in dispositional mindfulness (DM) tended to have less PTSS after a tornado. Nagy et al. (2020) reported that DM was associated with lower frequency of PTSD-related sleep disturbance. Thompson and Waltz (2010) identified that dispositional mindfulness, specifically non-judgment of experiences, accounted for a unique portion of the variance in PTSD avoidance symptoms. These studies provide support for the salutary effects of dispositional mindfulness on PTSS, but the mediating processes in this relationship have not been sufficiently analyzed.

Emotional exhaustion (EE) is the core component of burnout, which means “feelings of being overextended and depleted of one’s emotional and physical resources” (Leiter and Maslach, 2004). Emotion management is part of nurses’ job, especially for emergency nurses who have to confront a variety of sudden challenges in the work environment; long-term depletion of emotional resources can lead to EE, which decreases nurses’ abilities (Salvarani et al., 2019). Some studies suggested that PTSS contributes to understanding emotional exhaustion (Mealer et al., 2009; Ager et al., 2012; Katsavouni et al., 2016). However, according to the conservation of resources theory (COR), maintaining a stable, psychological well-being requires the conservation and growth of psychological resources; losing or lacking of gain of resources considered as sufficient for producing stress (Hobfoll, 1989). From this perspective, the lack of emotional resources required for a healthy mind may ultimately result in psychological disorders. Specifically, emotional exhaustion may be a consequence and a factor of PTSS.

Moreover, emotional exhaustion also seems to be related to dispositional mindfulness. Salvarani et al. (2019) found that emergency nurses with higher dispositional mindfulness showed lower emotional exhaustion levels. From a sample of 500 Chinese intensive care nurses, Lu et al. (2019) reported that disposition mindfulness moderates the effects of perceived stress on emotional exhaustion. Brown and Ryan (2003) suggested that individuals’ vitality, energy, and stress resistance are enhanced and preserved when practicing dispositional mindfulness. Considering the above-mentioned relationship between dispositional mindfulness and PTSS, it is worth identifying the investigative importance of emotional exhaustion’s role between dispositional mindfulness and PTSS among emergency nurses.

Coping styles may be another promising factor between dispositional mindfulness and PTSS. Coping is the engagement in psychological or behavioral responses to stress-inducing demands (Folkman and Moskowitz, 2004). It is commonly considered as an important factor that impacts psychological well-being and health; positive and negative coping (NC) are the most basic coping styles (Carnicer and Calderón, 2014). Positive coping (PC) can generate positive emotions and behaviors and assist individuals in identifying targeted resources for handling conceivable threats, so that the relationship between stress and negative outcomes is mitigated; NC impedes active coping efforts and exacerbates distress and is associated with greater emotional exhaustion and post-traumatic stress (Jesus et al., 2014; Burnos and Bargiel-Matusiewicz, 2018; Rodríguez-Rey et al., 2019). Lazarus and Folkman (1984) identified at least two broad categories of antecedents that directly influenced how people appraised and coped with situations: those linked to individual characteristics, and those linked to situational characteristics (Lazarus and Folkman, 1984). Numerous studies also demonstrated the significant relationship of dispositional mindfulness as a psychological trait and a coping style (Sriwilai and Charoensukmongkol, 2015; Keng and Tong, 2016; Huberty et al., 2018). Dispositional mindfulness facilitates individuals’ ability to generate positive appraisals and inhibits maladaptive responses to stressful situations (Garland et al., 2009). Nevertheless, we have not found evidence of coping styles as a mediator between dispositional mindfulness and PTSS in previous studies.

**Figure 1:**
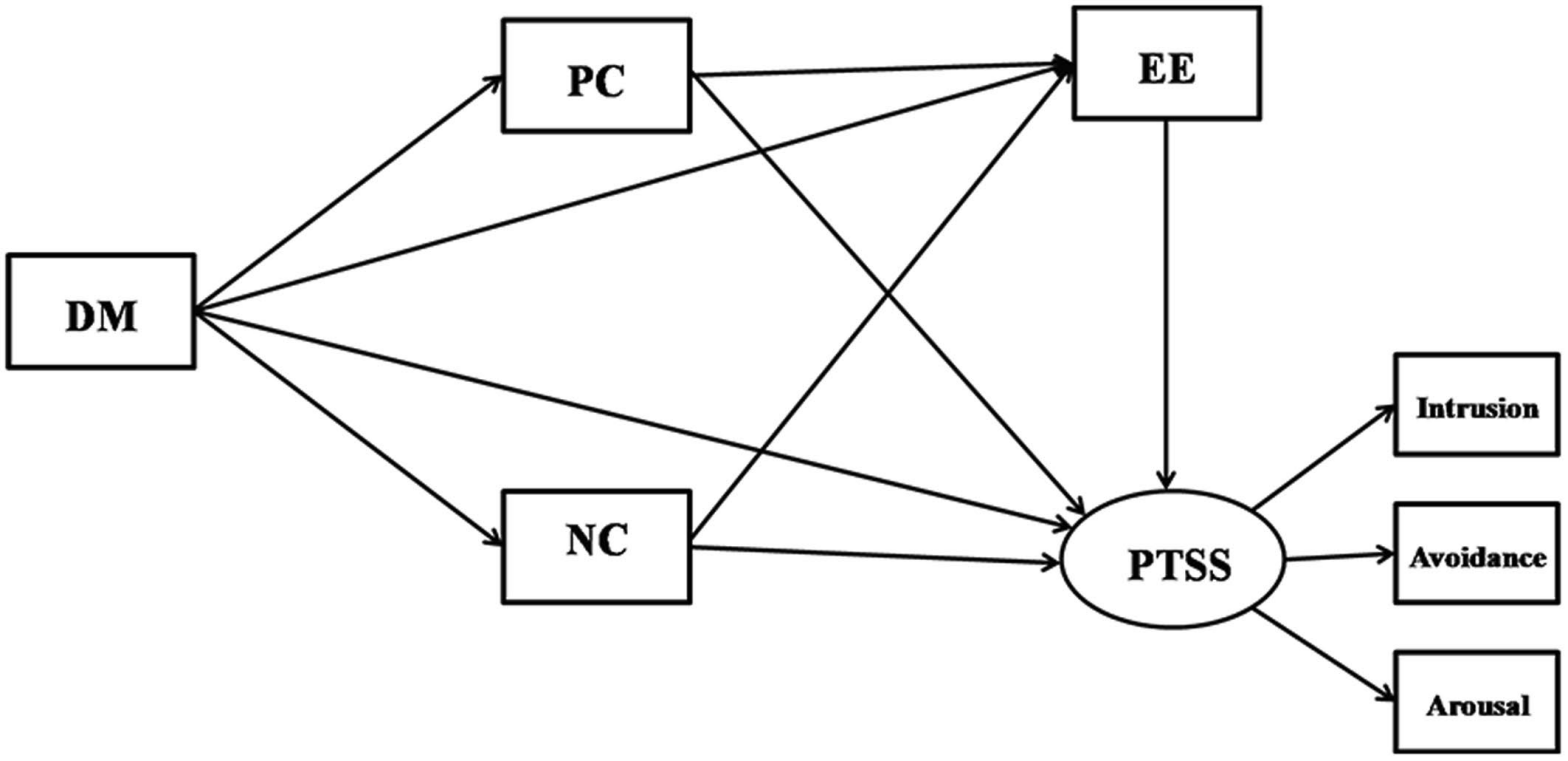
Hypothesized model of direct and indirect relationships between dispositional mindfulness and PTSS. DM, dispositional mindfulness; PC, positive coping; NC, negative coping; EE, emotional exhaustion; and PTSS, post-traumatic stress symptom.

As discussed above, emergency nurses’ dispositional mindfulness, coping styles, emotional exhaustion, and their relations to PTSS have been respectively examined. However, research focusing on these factors’ systematic effects on PTSS is still scant. Thus, the aim of this study was to highlight the specific process through which dispositional mindfulness impact emergency nurses’ PTSS and to investigate the multiple mediating effects of coping styles and emotional exhaustion on the relation between them. Specifically (as shown in Figure 1), we hypothesized that:

1. Dispositional mindfulness, coping styles, and emotional exhaustion directly predict PTSS.
2. Dispositional mindfulness indirectly predicts PTSS through the sequential mediating effect of coping styles and emotional exhaustion.
3. The effect of coping styles on PTSS is mediated by emotional exhaustion.

## Materials and Methods

### Participants

To be eligible, the participants must have worked in emergency department as registered nurses for more than 1 year. Nurses who were leaving their positions after more than 1 month were excluded. A total of 628 Chinese emergency nurses participated in this study; after excluding inaccuracies and incomplete data for the analyzed measures, 571 participants were included, with an effective response rate of 90.9%. The sample comprised of 517 (90.5%) females and 54 (9.5%) males; the majority were married 68.3%. They had an average age of 30.54 years (*SD* = 6.30), and the average working years of emergency nurses was 9.27 years (*SD* = 7.15). Approximately 74.3% of the participants had a bachelor’s degree.

### Measurement

#### Dispositional Mindfulness

Dispositional mindfulness was measured with the Chinese version of the Mindfulness Attention Awareness Scale (MAAS; Deng et al., 2012). MAAS was widely used to assess the awareness of the present moment and the level of attention. It had a single factor structure that included 15 items [scored from 1 (almost always) to 6 (almost never)]; higher scores indicated higher levels of dispositional mindfulness. It is a robust scale, with good reliability and internal consistency (Sriwilai and Charoensukmongkol, 2015). In this study, this instrument had a Cronbach’s alpha of 0.930, and indicated adequate structural validity: chi-square test of model fit (*χ*^2^) = 724.505, *df* = 300, *χ*^2^/*df* = 2.415, Comparative Fit Index (CFI) = 0.983, Tucker Lewis Index (TLI) = 0.970, and root mean squared error of approximation (RMSEA) = 0.050.

#### Post-traumatic Stress Symptoms

The Impact of Event Scale-Revised (IES-R) was used to assess the PTSS (Weiss, 2007). The scale is recognized as one of the earliest self-report tools developed to assess PTSD. It has 22 items and three subscales: intrusion, avoidance, and arousal, all items are rated on a five-point scale ranging from 0 (not at all) to 4 (extremely). Although this tool is not used for PTSD diagnosis (Wilson and Keane, 1996), a total score of 34 has been reported to predict clinical diagnosis of PTSD, and higher total scores indicate more severe PTSS (Adriaenssens et al., 2012; Sheen et al., 2015). A previous study demonstrated relatively high reliability and validity levels of the IES-R’s Chinese version in the Chinese population (Guan et al., 2019). In this study, the Cronbach’s alpha was 0.970, and the confirmatory factor analysis is as follows: *χ*^2^ = 682.522, *df* = 213, *χ*^2^/*df* = 3.204, CFI = 0.959, TLI = 0.951, and RMSEA = 0.052.

#### Emotional Exhaustion

Emotional exhaustion was examined using the emotional exhaustion subscale of the Maslach Burnout Inventory (MBI) developed by Maslach and Jackson (1981). The MBI has been widely used in previous studies, which was proven to be reliable and valid in China (Lu et al., 2019). The emotional exhaustion subscale consisted of nine items, and each item was evaluated from 0 “never” to 6 “every day”; higher scores correspond to higher frequencies of experienced emotional exhaustion. The scale demonstrated excellent, internal consistency (Cronbach’s alpha = 0.908) and acceptable structural validity (*χ*^2^ = 287.3, *df* = 80, *χ*^2^/*df* = 3.591, CFI = 0.986, TLI = 0.969, and RMSEA = 0.067) in the current sample.

#### Coping Styles

Simple Coping Style Questionnaire (SCSQ) is a well-established Chinese questionnaire compiled by Yaning (1988), which was used to measure participants’ coping style of life events in this study. It consisted of 20 items, including 12 items of PC eight items of negative coping. Each item was rated on a four-point scale, ranging from 0 (never) to 3 (always). The higher the one-dimensional scores, the more an individual tended to adopt this kind of coping style. The SCSQ has been shown to have good reliability and validity in a previous study (Gu et al., 2020). In this study, the Cronbach’s alpha coefficients of the positive coping and negative coping were 0.904 and 0.782, respectively. The confirmatory factor analysis indicated the following: *χ*^2^ = 1409.215, *df* = 605, *χ*^2^/*df* = 2.329, CFI = 0.964, TLI = 0.943, and RMSEA = 0.048, indicating adequate structural validity in our sample.

### Procedure

An anonymous online survey was conducted in Chongqing, China from July 30th to August 30th, 2019. Twenty hospitals were conveniently selected, using self-administered questionnaires to collect data on emergency nurses’ mindfulness, coping styles, emotional exhaustion, and PTSS. The link to our online questionnaire survey was sent to nurses (*via* the head nurses) after we received their approval to collect data for the study. The study’s objectives and contents and the participation’s voluntary nature were introduced on the questionnaire’s first page to ensure all participants were fully informed. The questionnaire completion and submission implied consent in joining the study. The survey was developed *via* an online platform Questionnaire Star;^1^ all data were numerically coded and accessible only to the researchers to protect confidentiality.

### Statistical Analysis

IBM® SPSS® Statistics (IBM Corp., Armonk, NY) 22.0 was used to analyze the data, and all statistical tests were two-sided (*α* = 0.05). Spearson’s correlation analysis was used for exploring the relationship between dispositional mindfulness, coping styles, emotional exhaustion, and PTSS. The mediating effects were tested with the AMOS24.0; structural equation modeling (SEM) was executed to test the multiple mediation model. Before performing the regression analyses, all continuous variables were centralized in order to avoid multicollinearity. The control variables (age and education background) were used, because they were assumed to be related to PTSS. Goodness of fit was evaluated by several indices: *χ*^2^, the CFI, the TLI, and the RMSEA. In this study, the model could be considered as an acceptable fit when: *χ*^2^/*df* was below 5, and the CFI and TLI values were above 0.90, whereas RMSEA values of 0.05 or lower. Furthermore, a bootstrap estimation procedure with 2,000 bootstrap samples was conducted to examine the mediating hypotheses (Preacher and Hayes, 2008). For each independent variable, when the bias-corrected and accelerated 95% CI of indirect effect excluded 0, it was concluded that the mediating effect was statistically significant.

## Results

## PTSS Status

Approximately two-fifths of the emergency nurses who participated (*n* = 230, 40.3%) exceeded the cut-off score, indicative of symptoms that were commensurate with a clinical diagnosis of PTSS. The average total score of IES-R was 28.57 ± 17.93; the highest mean score (total score/items) of the three dimensions was intrusion (1.39 ± 0.89), followed by arousal (1.27 ± 0.86) and avoidance (1.26 ± 0.82).

### Means, SD, Correlations Among Study Variables

Results of means, SDs, and Spearson correlation analysis are shown in Table 1. Significant correlations were observed between all study variables. PTSS was significantly and positively related to negative coping and emotional exhaustion (*r* = 0.254 ~ 0.426, *p* < 0.05); PTSS was significantly and negatively related to dispositional mindfulness and positive coping (*r* = −0.115 ~ −0.409, *p* < 0.05). Higher dispositional mindfulness was associated with less negative coping (*r* = −0.153, *p* < 0.05), more positive coping (*r* = 0.278, *p* < 0.05), and lower levels of emotional exhaustion (*r* = −0.343, *p* < 0.001).

**Table 1:** Means, SDs, and Spearson’s correlations among study variables (*n* = 571).

Variable	***M* ± ** ***SD***	**1**	**2**	**3**	**4**	**5**	**6**	**7**	**8**
1. Dispositional mindfulness	55.64 ± 14.47	1.000							
2. Positive coping	22.71 ± 6.75	0.278^***^	1.000						
3. Negative coping	10.46 ± 4.24	−0.153^***^	0.248^***^	1.000					
4. Intrusion	8.32 ± 5.33	−0.359^***^	−0.095^*^	0.241^***^	1.000				
5. Avoidance	10.09 ± 6.57	−0.385^***^	−0.084^*^	0.233^***^	0.871^***^	1.000			
6. Arousal	10.15 ± 6.85	−0.418^***^	−0.143^**^	0.259^***^	0.861^***^	0.846^***^	1.000		
7. PTSS total	28.57 ± 17.93	−0.409^***^	−0.115^**^	0.254^***^	0.948^***^	0.950^***^	0.952^***^	1.000	
8. Emotional exhaustion	20.90 ± 11.78	−0.343^***^	−0.210^***^	0.238^***^	0.391^***^	0.371^***^	0.439^***^	0.426^***^	1.000

*M, mean and SD, standard deviation*.

* *p* *< 0.05;*

** *p* *< 0.01;*

*** *p < 0.001*.

### Structural Model

**Figure 2:**
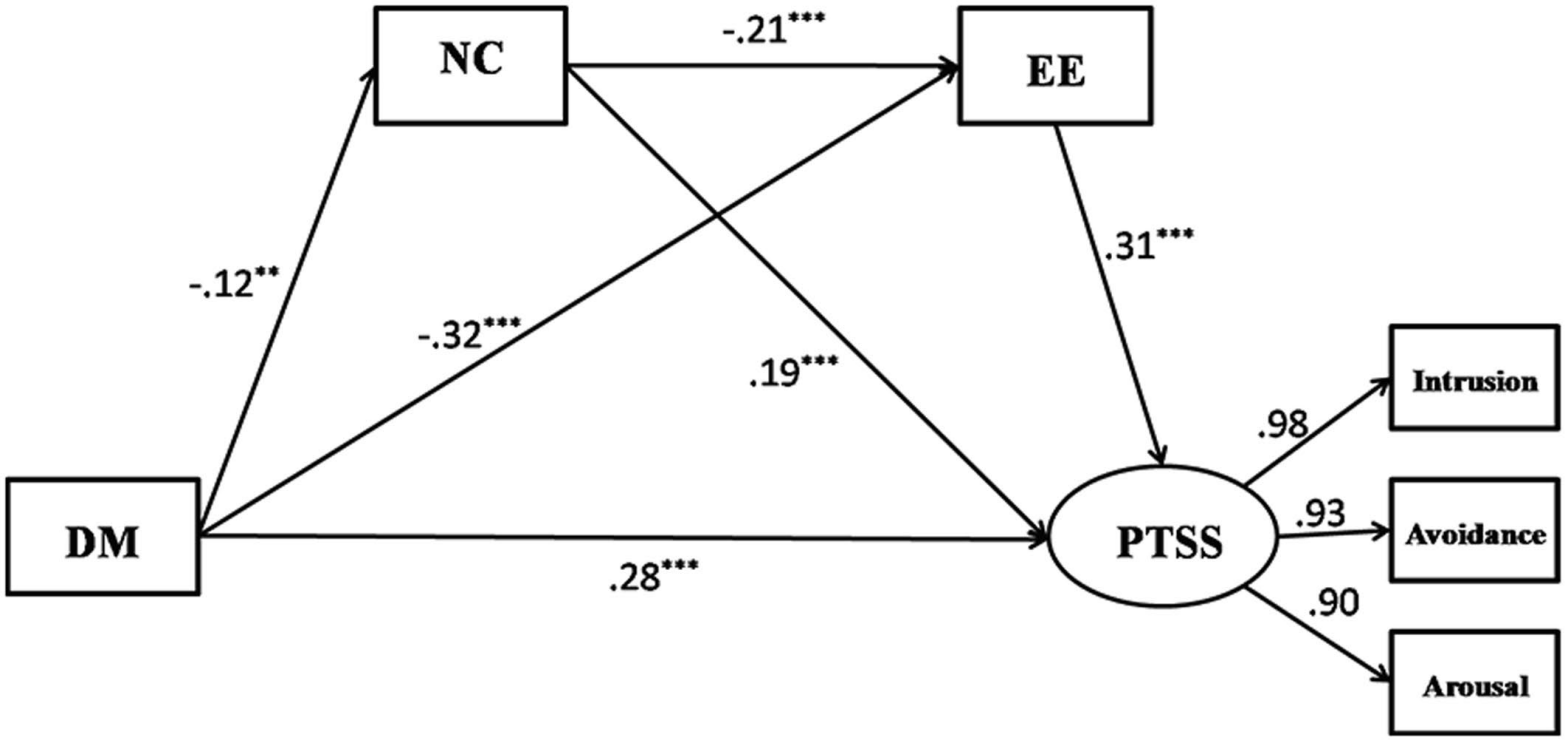
Path coefficient estimates of the modified model (M0): Direct and indirect effect of dispositional mindfulness on PTSS through negative coping and emotional exhaustion. DM, dispositional mindfulness; NC, negative coping; EE, emotional exhaustion; and PTSS, post-traumatic stress symptoms. ^**^p < 0.01 and ^***^p < 0.001.

The results of the SEM analysis showed that the goodness of fit for the hypothesized model was: *χ*^2^ = 60.915, *df* = 20, *p* < 0.01, *χ*^2^/*df* = 3.046, CFI = 0.982, TLI = 0.968, and RMSEA = 0.060. Path consideration from positive coping to PTSS was not statistically significant (*β* = −0.03, *p* > 0.05), and positive coping did not have a significant, mediating role between dispositional mindfulness and PTSS (*β* = −0.009, 95% CI: −0.037 ~ 0.017); we removed the positive-coping variable and formed a modified model (M0). Values of fit indices indicated an excellent model fit: *χ*^2^ = 5.784, *df* = 4, *p* < 0.01, *χ*^2^/*df* = 1.446, CFI = 0.999, TLI = 0.997, and RMSEA = 0.028, as shown in Figure 2.

**Figure 3:**
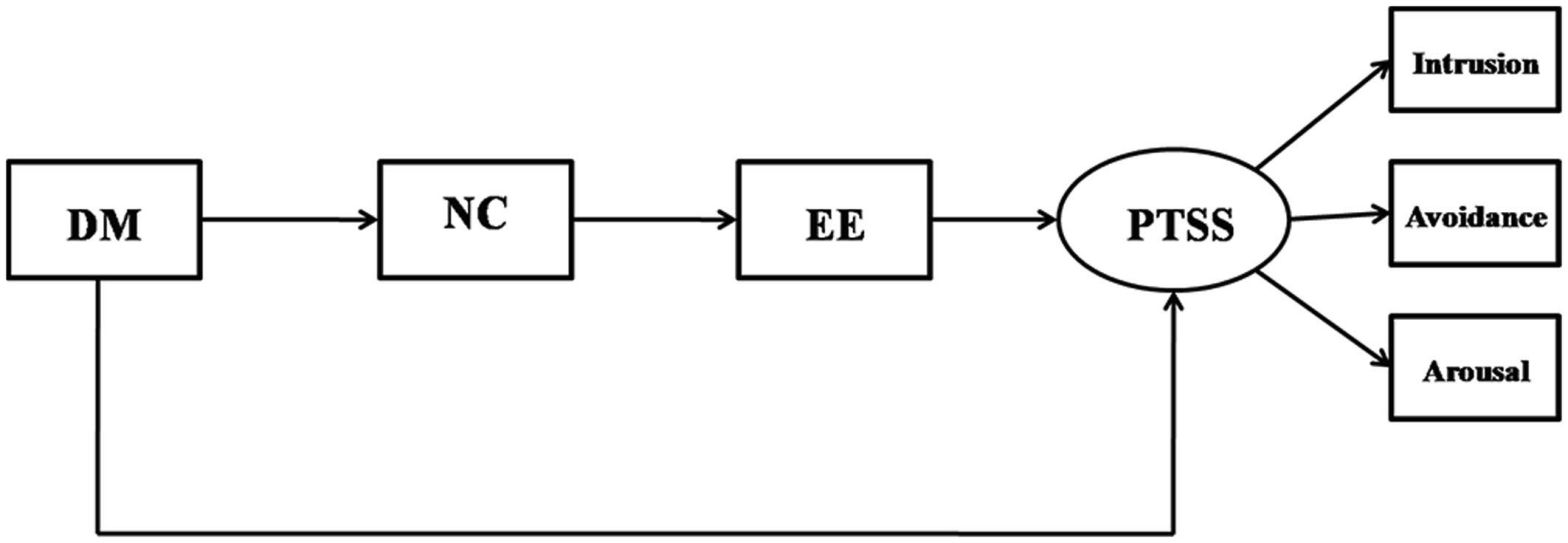
The competing model M1 (sequential mediation model): Dispositional mindfulness affects emotional exhaustion through negative coping and then to PTSS. DM, dispositional mindfulness; NC, negative coping; EE, emotional exhaustion; and PTSS, post-traumatic stress symptoms.

**Figure 4:**
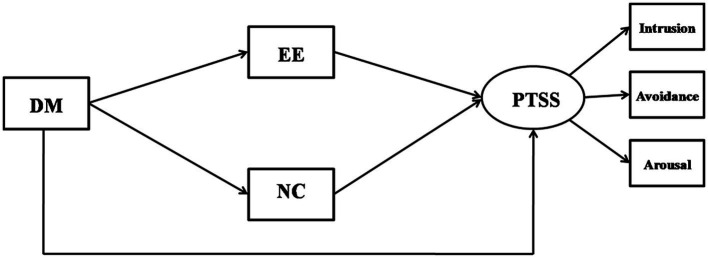
The competing model M2 (parallel mediation model): Negative coping and emotional exhaustion as parallel mediators between dispositional mindfulness and PTSS. DM, dispositional mindfulness; NC, negative coping; EE, emotional exhaustion; and PTSS, post-traumatic stress symptoms.

To test the modified model and to determine the relationship between negative coping and emotional exhaustion, two competing models were proposed. Competing model M1 removed the path from dispositional mindfulness to emotional exhaustion and path from negative coping to PTSD symptoms, forming a sequential mediation model (shown in Figure 3). Competing model M2deleted the paths from negative coping to emotional exhaustion and formed a parallel mediation model (shown in Figure 4). The goodness of fit assessment demonstrated that the model fit for M1 (*χ*^2^ = 114.097, *df* = 8, *χ*^2^/*df* = 14.262, *p* < 0.01, CFI = 0.949, TLI = 0.905, and RMSEA = 0.153) and M2 (*χ*^2^ = 52.537, *df* = 7, *p* < 0.01, *χ*^2^/*df* = 7.505, CFI = 0.978, TLI = 0.953, and RMSEA = 0.107) were not adequate as shown by the indicators. The modified model indicated an adequate fit in comparison.

### Test for Mediation

As shown in Table 2, all standardized, indirect coefficients of dispositional mindfulness on PTSS were statistically significant, which supported our hypothesis that coping style and emotional exhaustion had concurrent and sequential mediating effects on dispositional mindfulness and PTSS. Specifically, the relationship between dispositional mindfulness and emotional exhaustion was partly mediated by negative coping (*β* = −0.026, *p* < 0.05), and the influence of negative coping on PTSS was partly mediated by emotional exhaustion (*β* = 0.065, *p* < 0.05). Negative coping and emotional exhaustion severally mediated the effect of dispositional mindfulness on PTSS (negative coping: *β* = −0.024; emotional exhaustion: *β* = −0.098); they also sequentially mediated the relationship between dispositional mindfulness and PTSS in a significant manner (*β* = −0.008, *p* < 0.05), which was consistent with our hypothesis.

**Table 2:** Effect estimates of the modified model.

Structural paths	Direct effect (*β*)	Indirect effect	
		Paths	*β*	95% CI lower, upper	Standardized total effect
DM→NC	−0.124^*^	-	-	-	−0.124^*^
DM→EE	−0.320^***^	DM→NC→EE	−0.026^*^	−0.054, −0.003	−0.346^***^
DM→PTSS	−0.278^***^	DM→NC→PTSS	−0.024^*^	−0.048, −0.003	−0.408^***^
		DM→EE→PTSS	−0.098^***^	−0.136, −0.065	
		DM→NC→EE→PTSS	−0.008^*^	−0.017, −0.001	
NC→PTSS	0.192^***^	NC→EE→PTSS	0.065^**^	0.031, 0.102	0.257^***^
EE→PTSS	0.305^***^	-	-	-	0.305^***^

*DM, dispositional mindfulness; NC, negative coping; EE, emotional exhaustion; and PTSS, post-traumatic stress symptoms*.

* *p* *< 0.05;*

** *p* *< 0.01;*

*** *p < 0.001*.

## Discussion

We developed a multiple-mediation model between dispositional mindfulness and PTSS and tested the mediating roles of coping styles and emotional exhaustion based on studies that explored PTSS’s influencing factors among emergency nurses. Through multiple models’ comparison, our hypothesized mediation model proved largely accurate, and the results supported most of the hypotheses.

As expected, all variables were correlated. Emergency nurses with higher dispositional mindfulness had lower PTSS and emotional exhaustion, which was consistent with previous reports that dispositional mindfulness had an important protective role in mental health, regardless of the traumatic experience (Thompson and Waltz, 2010; Lu et al., 2019; Meyer et al., 2019). Additionally, our results showed that emergency nurses, who preferred negative coping and experienced more emotional exhaustion, reported higher PTSS, which supported previous conclusions that highlighted negative coping’ positive role in PTSS; the results also confirmed the argument that emotional exhaustion’s role in PTSS development has been overlooked to some extent in the past (Burnos and Bargiel-Matusiewicz, 2018; Jo et al., 2018; Kucmin et al., 2018; Slade et al., 2020).

The current findings expanded upon prior studies, we found that negative coping and emotional exhaustion had mediating roles between dispositional mindfulness and PTSS, separately. In other words, dispositional mindfulness not only directly affected PTSS, but also indirectly influenced PTSS through decreasing negative coping strategies and emotional exhaustion. This finding provides two new targets, i.e., negative coping and emotional exhaustion, in the PTSS coping of emergency nurses. As the model of COR (Hobfoll, 1989) suggests individuals can employ other resources to offset net loss after stress events. Support programs that increase psychological resources are necessary for exhausted nurses. Mindfulness can be used to increase individuals’ psychological ability to manage the external pressures’ psychological impact, improve the emotion regulation’s ability, decrease emotional depletion, and prevent the PTSS that is caused by emotional exhaustion (Hülsheger et al., 2013; Tang et al., 2016). Additionally, mindfulness can also be used to help with cognitive reappraisal, which allows for emergency nurses to appraise situations (in a composed manner) with less stress and less use of maladaptive styles when coping with stress (Keng and Tong, 2016; Rodríguez-Rey et al., 2019; Gu et al., 2020). Unlike other personality factors, such as neuroticism, which are hard to change, dispositional mindfulness can be reinforced with practice. Recently, studies in which researchers investigated mindfulness-based interventions have substantially increased, which have shown significant improvement in mindfulness for facilitating coping capacity and treating various mental outcomes (e.g., PTSD, anxiety, etc.) among clinical and non-clinical populations (Polusny et al., 2015; Hopwood and Schutte, 2017; Sarenmalm et al., 2017). Some researchers had applied mindfulness-based training to clinical nurses and found that even a brief intervention was conducive to stress management (Mackenzie et al., 2006), which indicated that routine mindfulness exercises can be promising methods for boosting immunity to trauma in nurses. Other specific activities that reduce negative coping and emotional exhaustion are also necessary. A previous study found the significant effects of work overload, work shift, and age on emotional exhaustion among emergency nurses (Li et al., 2018). Another work found that nurses who worked night shifts were more likely to use confrontation strategies (Ribeiro et al., 2015). Such evidence indicated that sufficient attention should be given to younger nurses who frequently work night shifts and have less supervisory support. Scientific scheduling system, a harmonious organizational atmosphere, rational allocation of human resources, and programs that have been confirmed to improve emotion regulation and coping capacity, such as coping skills, training, and regular psychological guidance, should also be considered by organizations (Medisauskaite and Kamau, 2019; Guo et al., 2020).

Unexpectedly, we did not obtain results for positive coping. Such finding contrasts with previous research indicating that positive coping has a significant effect on PTSS (Britt et al., 2017; Baral and Bhagawati, 2019). As correlation analyses have shown, positive coping was significantly related to PTSS; however, the relationship became non-significance when other variables were introduced in the model. Negative coping was a much stronger predictor of PTSS than positive coping, indicating that mindfulness exerts an impact on PTSS, primarily through reducing negative coping strategies, which was coherent with previous studies (Read et al., 2014; Theleritis et al., 2020). This data may be explained by the fact that although negative coping strategies may reduce psychological stress in the short term, it may lead to insufficient social support, which in turn, increases chronic stress and psychological tension in the long run; PTSS may then be exacerbated (Carver et al., 1989; Cheng et al., 2020). Consistent with our hypothesis, negative coping and emotional exhaustion also sequentially mediated dispositional mindfulness and PTSS. In other words, negative coping not only directly affected PTSS but also aggravated emotion-resource depletion and ultimately, increased stress. Thus, it may be more important for individuals to avoid negative coping strategies when dealing with stress.

Dispositional mindfulness is beneficial for decreasing PTSS and the impact of emotional exhaustion and negative coping strategies on PTSS. These findings highlighted a central mechanism that underlined mindfulness’ salutary effects on the mental health of emergency nurses, revealed other possible pathways for explaining the relationship between dispositional mindfulness and PTSS, and provided a new theoretical basis and methods for preventing mental disorder in emergency nurses. Primary screening and preventive efforts should be performed to identify emergency nurses who have emotional exhaustion and those who prefer negative coping strategies. Additionally, mindfulness-based interventions for emergency nurses should be considered and incorporated at work, which may facilitate their ability to constructively cope (other than focus on negative coping strategies) with stress and reduce emotional exhaustion.

## Limitation

This study should be interpreted in light of its limitations. First, the cross-sectional design used in the study precluded causal relationships and strongly limited our ability to infer causality. Longitudinal studies are needed to further examine the relationships among the variables in greater depth. Second, the convenience sampling strategy to enroll participants could not obtain representative samples. Further work using larger samples from multicenter and across regions could help us to develop a better understanding of occurrence and development mechanisms of PTSS in emergency nurses. Third, all variables were measured using self-report scales. Although the measures were common in trauma studies, there is a risk of consistency self-rating bias and social desirability. A survey combined with semi-structured interview may produce more comprehensive results.

## Conclusion

To sum up, dispositional mindfulness, emotional exhaustion, and coping styles were strongly associated with PTSS in emergency nurses. Dispositional mindfulness has the potential to act as a stress-coping resource, affecting individuals’ post-traumatic stress outcome by reducing emotional exhaustion and by influencing coping strategies, while dealing with stressful working conditions. Mindfulness, emotional exhaustion, and negative coping constitute critical factors in organizational intervention strategies aimed at preventing PTSS among emergency nurses.

## Data Availability Statement

The raw data supporting the conclusions of this article will be made available by the authors, without undue reservation.

## Author Contributions

YY and WS: conceptualization. WS: supervision. YS, XX, FL, and FX: investigation. YY: formal analysis, data curation, funding acquisition, and original manuscript draft. YY and ZW: manuscript review and editing. All authors contributed to the article and approved the submitted version.

## Funding

This study was partially supported by the Project of Humanities and Social Science Foundation of Army Medical University (Project No. 2019XRW12).

## Conflict of Interest

The authors declare that the research was conducted in the absence of any commercial or financial relationships that could be construed as a potential conflict of interest.

## Publisher’s Note

All claims expressed in this article are solely those of the authors and do not necessarily represent those of their affiliated organizations, or those of the publisher, the editors and the reviewers. Any product that may be evaluated in this article, or claim that may be made by its manufacturer, is not guaranteed or endorsed by the publisher.

## Footnotes

^1^ https://www.wjx.cn/
